# Supplementary material for: Chromothripsis during telomere crisis is independent of NHEJ, and consistent with a replicative origin
Source: Genome Res. 2019 May;29(5):737–49. doi: 10.1101/gr.240705.118 (PMC6499312; doi:10.1101/gr.240705.118)
Supplement: Supplemental Material [file supp_gr.240705.118_Supplemental_file_1.zip › contigs/annotated_contigs/DB67/contig.2.DB67_length_315_mean_cov_5.63174603175.docx]

**DB67_length_315_mean_cov_5.63174603175**

```
GTGCTGGAGGCACCAAATAGAGTCTGCTCTATACATCTTTTTACAATTTTTAAAA|ATTATTATTATTATTATTATTATTATTATT|TG
 >chr7:72789701-72789786 - E=9e-40 p=0e+00              >chr7:72789455-72789710 - E=3e-
AGACGGAGTTTTGTTCTTGTTGCCCAGGCTGGAGTGCAATGGCACGATCTCGGCTCACCGCAACCTCCGCCTCCCAGATTTAAGCGATT
112
CTCCTGCCTCAGCCTACCGAGTAACTTGGAATACAGGCATGTGCCACCACGCCTGGCTAATCTTGAAGTTTGAGTAGAGACAGGGTTTC

TCCATGGGGGTCCGGCTGCCCTTGCACCCCTGAAGTCAGGTGATC|CGCCC
```
